# Protective Role of Apigenin Against Aβ 25–35 Toxicity Via Inhibition of Mitochondrial Cytochrome c Release

**DOI:** 10.32598/BCN.9.10.385

**Published:** 2019-11-01

**Authors:** Farnaz Nikbakht, Yasaman Khadem, Sobhan Haghani, Hadiseh Hoseininia, Alireza Moein Sadat, Paria Heshemi, Nida Jamali

**Affiliations:** 1. Cellular and Molecular Research Center, Iran University of Medical Sciences, Tehran, Iran.; 2. Department of Physiology, School of Medicine, Iran University of Medical Sciences, Tehran, Iran.

**Keywords:** Apigenin, Alzheimer, Memory, Cytochrome c

## Abstract

**Introduction:**

Cognitive dysfunction is the most common problem of patients with Alzheimer Disease (AD). The pathological mechanism of cognitive impairment in AD may contribute to neuronal loss, synaptic dysfunction, and alteration in neurotransmitters receptors. Mitochondrial synapses dysfunction due to the accumulation of Amyloid Beta (Aβ) is one of the earliest pathological features of AD. The flavone apigenin has been reported to play some protective roles in AD through the anti-oxidative and anti-inflammatory properties. This study aimed at investigating the effects of apigenin on spatial working memory and neural protection by restoring mitochondrial dysfunction and inhibition of caspase 9.

**Methods:**

Intracerebroventricular (ICV) microinjection of Aβ 25–35 was used for AD modeling. Working memory was assessed 21 days later using the Y maze test. Neuronal loss was detected in the hilar area of the hippocampus using Nissl and Fluoro-jade B staining, whereas immunohistochemistry was used to illustrate cytochrome c positive cells and caspase 9.

**Results:**

The results revealed that apigenin significantly ameliorated spatial working memory. It also significantly reduced the number of degenerative neurons in the hilus area. Apigenin almost completely blocked the release of cytochrome c and caspase 9 in hilus.

**Conclusion:**

Apigenin may improve the spatial working memory deficits and neuronal degeneration through the amelioration of the mitochondrial dysfunction.

## Highlights

- Apigenin ameliorated spatial working memory impairment induced by Aβ 25–35.
- Apigenin protected hilar neuronal loss induced by Aβ 25–35.
- Apigenin inhibited mitochondrial apoptotic pathways induced by Aβ 25–35.

## Plain Language Summary

Memory problem is the main complaint of patients with Alzheimer’s disease. According to previous studies some herbal remedies can improve memory deficits in AD. This study evaluates the beneficial effects of flavone apigenin from celery on memory restoration. According to the results, treatment with apigenin has improved the spatial memory problems in AD which has been accompanied by a protection in mitochondria.

## 1. Introduction

Alzheimer Disease (AD) is the most common type of senile dementia, which is characterized by the irreversible loss of neurons and synapses in the brain (Zhu et al., 2013). The exact underlying mechanism of vast neurodegeneration in AD is unclear; however, many studies have suggested that a mitochondrial abnormality is an early event in AD since mitochondrial dysfunction is detectable in neurons lacking neurofibrillary tangles (Du et al., 2010; Hu & Li, 2016; Reddy, 2007). Accumulation of Amyloid Beta (Aβ) in mitochondria by interacting with mitochondrial proteins leads to the release of cytochrome c, which in turn can activate the caspase cascade (Pavlov, Petersen, Glaser, & Ankarcrona, 2009). Apparently, there is an amplification feedback loop between mitochondria dysfunction, cytochrome c release, and caspase activation.

Neuronal loss is a late manifestation of exposure to Aβ, which inevitably is a major cause of the cognitive deficit. However, mitochondria and synaptic dysfunction have been found to occur before neuronal cell death in Aβ models, which is the cause of memory impairment (D’amelio et al., 2011; Ford et al., 2015). Therefore, early-stage disruptions of mitochondria are excellent targets for the treatment of AD. Recently, there has been considerable interest in the therapeutic potential of flavonoids in degenerative diseases (Solanki, Parihar, Mansuri, & Parihar, 2015).

Flavonoids possess neuroprotective properties, which are involved in several brain processes, including neuronal protection against injury and an increase in memory, learning, and cognitive function (Devi & Ohno, 2012). It has been shown that these dietary compounds have some effect on mitochondrial function and dynamics, as well (Serrano Casasola, Cassanyé, Martín-Gari, Granado-Serrano, & Portero Otín, 2016). Therefore, flavonoids can be used as the key molecules for the treatment of AD.

Apigenin (4′,5,7-trihydroxyflavone) is a flavonoid, particularly abundant in the chamomile plant (68% apigenin of total flavanoids) (McKay & Blumberg, 2006) and is found in lower concentrations in other sources, such as celery, parsley, and grapefruit (Shukla & Gupta, 2010). Apigenin is a potent antioxidant, anti-inflammatory, and anti-carcinogenic agent. Also, some other studies have shown the beneficial effects of apigenin on AD (Zhao et al., 2011; Zhao, Wang, Liu, et al., 2013; Zhao, Wang, Wang, & Fa, 2013). It can improve the memory deficit induced by Aβ 25–35 through the restoration of the Brain-derived Neurotrophic Factor (BDNF) pathways, a key molecule involved in learning and memory (Zhao, Wang, Liu, et al., 2013).

Despite some investigations on apigenin protective effects due to its interaction with Aβ peptides (Zhao, Wang, Wang, & Fa, 2013; (Zhu et al., 2013), its therapeutic effects on Aβ-induced cognitive impairment and its relation to mitochondrial dysfunction has not well been studied. Thus, this study aimed at evaluating the anti-amnesic potential of apigenin regarding its beneficial effect on the inhibition of cytochrome c release, caspase activation, and hippocampal cell protection.

## 2. Methods

### 2.1. Reagents

Aβ 25–35 (Sigma A4559) and sodium carboxymethyl cellulose (Sigma 419273) were purchased from the Sigma Chemical Co. (Saint Louis, Missouri USA). Fluoro-Jade B (AG310) was obtained from the Millipore Company (Billerica, Massachusetts, USA). Apigenin (98% tested by HPLC; 520-36-5) was prepared from the Shaanxi Huike Botanical Ltd (China). Aβ was dissolved in saline and microinjected into the Ieft lateral Ventricle (ICV) at a final concentration of 15 nmol/5 μL.

Apigenin was dissolved in distilled water containing 5% sodium Carboxyl Methylcellulose (CMC-Na) at a concentration of 10 mg/mL. Apigenin-treated rats received apigenin by oral gavage every day at a dose of 50 mg/kg body weight. The control or vehicle group received 5% CMC-Na orally during the experimental period. The treatment process lasted for 4 weeks.

The following antibodies were used for immunohistochemistry: rabbit polyclonal antibody to caspase 9 (Biorbyt, Cat. orb10242) diluted 1:100, rabbit anti-rat cytochrome c primary antibody (Biorbyt, Cat. Orb10508) diluted 1:100, and goat anti-rabbit IgG secondary antibody (Biorbyt, cat. Orb43514) diluted 1:100.

### 2.2. Animals

This study was conducted in the experimental animal house, Iran University of Medical Sciences, Tehran, Iran. Fifty adult male Wistar rats weighing 200–250 g were kept (four per cage) at room the temperature of 21±2º C with a 12/12h light/dark cycle. Rats were given ad libitum access to food and water. All procedures on animals were reviewed and approved by the Institutional Animal Care and Use Committee of the Tehran University of Medical Sciences (IACUC protocol number: [93-02-45-26666]).

### 2.3 Experimental design

Animals were randomly divided into 5 groups: control/vehicle, apigenin, Aβ, apigenin+Aβ, and sham-operated. Due to no significant differences in the analysis of sham and vehicle groups, the data of control and sham groups were pooled together.

### 2.4. Operation

After deep anesthesia with (100 mg/kg of ketamine and 7 mg/kg of xylazine IP), rats were placed in the stereotaxic apparatus (Stoelting, USA) and unilaterally microinjected aggregated (48 h 37º) Aβ 25–35 into the left lateral ventricle, according to the coordinates of Paxinos: AP: −0.8, L: +1.5, and DV: −3.4 (Paxinos, 2007).

### 2.5. Behavioral test

Three weeks after ICV microinjection of Aβ, the Y maze task was performed. One week later, the animals were sacrificed, and their brains were used for histological processes.

### 2.6. Y maze task

Spatial working memory in rats was evaluated with the Y maze test. Comparing to other spatial memory tests, the natural tendency of rodents to explore new environments serves as the basis of the Y maze task. The operator was blinded to the treatment received by the rats. Three weeks after ICV injection of Aβ, each rat was placed at the end of one arm and allowed to explore the apparatus for 8 min freely. The sequence and number of all arm entries were recorded for each animal throughout the period. Alternation rate was defined as entries into all three arms on consecutive occasions using the following Formula 1:

1. Alteration rate (%)=Number of alterations/(number of total arm entries-2)×100

### 2.7. Histology

Thirty days after AD modeling by ICV injection of Aβ, rats were deeply anesthetized with ketamine (100 mg/kg) and xylazine (7 mg/kg IP) and perfused through the ascending aorta first with 0.9% sodium chloride for 5 min followed by 4% paraformaldehyde in 0.1 M phosphate buffer (pH 7.4). Brains were post-fixed overnight at 4° C, removed, and embedded in paraffin. Serial microtome sections from the hippocampus area were mounted onto gelatin-coated slides and stained with Nissl and Fluoro-Jade B stains, cytochrome c, and caspase 9 (immunohistochemistry). As hilus was the best-protected area in the hippocampus in apigenin-treated rats, we only presented the data related to this area.

#### 2.7.1. Nissl staining

Coronal sections of the dorsal hippocampus with 5-μm thickness were Nissl-stained (0.1% cresyl violet acetate). At least 10 sections of each brain were counted and analyzed. The mean number of dark necrotic pyramidal cells in a total area of 431.637 mm ^2^ of hilus was expressed as the total count obtained from the representative sections. Counters were blind to the treatments. All sections were visually inspected using an Olympus light microscope (with the magnification of 200×) using OLYSIA Bio Report Soft Imaging System GmbH, V. 3.2 (Build 670).

#### 2.7.2. Fluoro-Jade B immune-florescent staining

The staining protocol was used as described in detail by Schmued, LC (Schmued & Hopkins, 2000). Briefly, the section (5 μm) of the brain were immersed in xylene and rehydrated by different alcohol solutions. The slides were then placed into a solution of 0.06% potassium permanganate for 10 min. After that, the slides were incubated for 30 min in the Fluoro-Jade B staining working solution (0.0004% with 0.1% acetic acid) in the dark. After three washes in distilled water for 1 min each, the sections were dried completely, immersed in xylene, and mounted with DPX. The tissue was examined using an epifluorescence microscope with blue (460–500 nm) excitation light. Fluoro-Jade B positive cells were counted from five sections per brain through the hilus area.

#### 2.7.3. Immunohistochemistry

The sections (5 μm) cut for immunohistochemical staining were first left for 60 min at 60º C, then deparaffinized in xylol for 20 min, and rehydrated by a successive series of alcohol. The sections were left for 20 min in 3% H _2_ O _2_ solution for blocking endogenous peroxidase. After washing with PBS, they were heated indirectly for 30 min in Bain Marie (70º C) and then left in blocking serum for 20 min. The sections were incubated overnight at 4ºC with primary antibodies at dilutions (1:100) (anti-cytochrome c and anti-caspase 9 rabbit polyclonal antibody). An HRP-conjugated goat anti-rabbit IgG was used as a secondary antibody. The sections were incubated for 60 min at room temperature. Chromogenic 3, 3′-diaminobenzidine (DAB) solution was used for detecting cells, and also Mayer’s hematoxylin (Thermo Scientific Inc. Waltham, MA, USA) was used for counterstaining. Caspase 9 or cytochrome c-positive cells were counted through the whole area of hilus.

### 2.8 Statistical analysis

Values are expressed as Mean±SEM. One-way ANOVA and Tukey’s post hoc test were used to assess the significance of differences between groups in Y maze and histological analysis. P<0.05 was considered statistically significant.

## 3. Results

### 3.1. Behavioral tests

#### 3.1.1. Apigenin treatment and spatial working memory deficit induced by Aβ 25–35 in Y maze test

**Figure 1.**
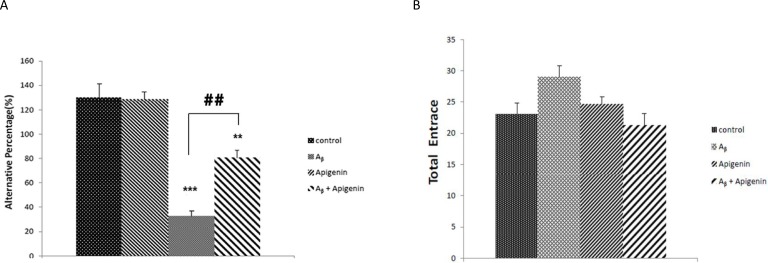
A. Apigenin treatment ameliorated spatial working memory impairment following intracerebroventricular injection of Amyloid-Beta (Aβ) in the Y maze task. The arm entry alterations decreased in the Aβ group (P<0.001 compared with the control group). However, apigenin treatment significantly ameliorated the working memory impairment in the Aβ group (P<0.01 Aβ vs. Aβ+apigenin), n=8. B. The total number of arm entries in the Y maze test through 8 min. No difference in motor activity was observed in groups (n=8), suggesting the normal activity of animals in all groups.

To show the effect of apigenin on cognitive preservation, we first examined the performance of the rats in the alternation Y maze task--a sensitive test to measure spatial working memory. Twenty-one days after Aβ injection, the rats in the Aβ group showed reduced spontaneous alternations compared with the control group [(Figure 1 A, (F _3,31_ =92.616, P<0.001)], suggestive of hippocampal dysfunction. However, such dysfunction was ameliorated after apigenin treatment [(Figure 1A), P<0.01)]. No difference was observed in the total arm entries between groups, indicating the normal activity of animals (Figure 1 B).

#### 3.1.2. Apigenin treatment and hilus cells loss induced by Aβ 25–35

**Figure 2.**
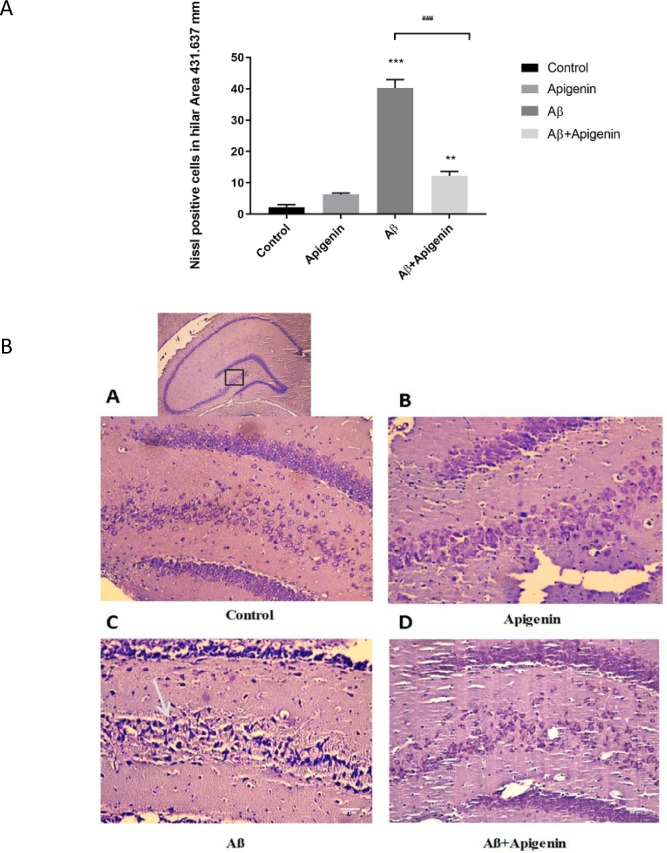
A. Representative bars of quantitative analysis of the necrotic cells of the hilus area. Apigenin significantly ameliorated the necrotic effect of Amyloid Beta (Aβ) in hilus (P<0.001 compared with Aβ group). Values are presented as Mean±SE (n=4). ^***^ P<0.001 and ^**^ P<0.01 vs. control group; ^###^ P<0.001 vs. Aβ group. B. a. Representative photomicrographs of Nissl-stained brain sections of the hilus area of the control; b. Apigenin; C. Amyloid beta (Aβ); and D. Aβ+apigenin groups. The lower magnification is presented above. Severe cell loss is evident in the Aβ group (arrow), which remarkably conserved after apigenin treatment. Scale bar for A–D: 50 μm (n=4).

**Figure 3.**
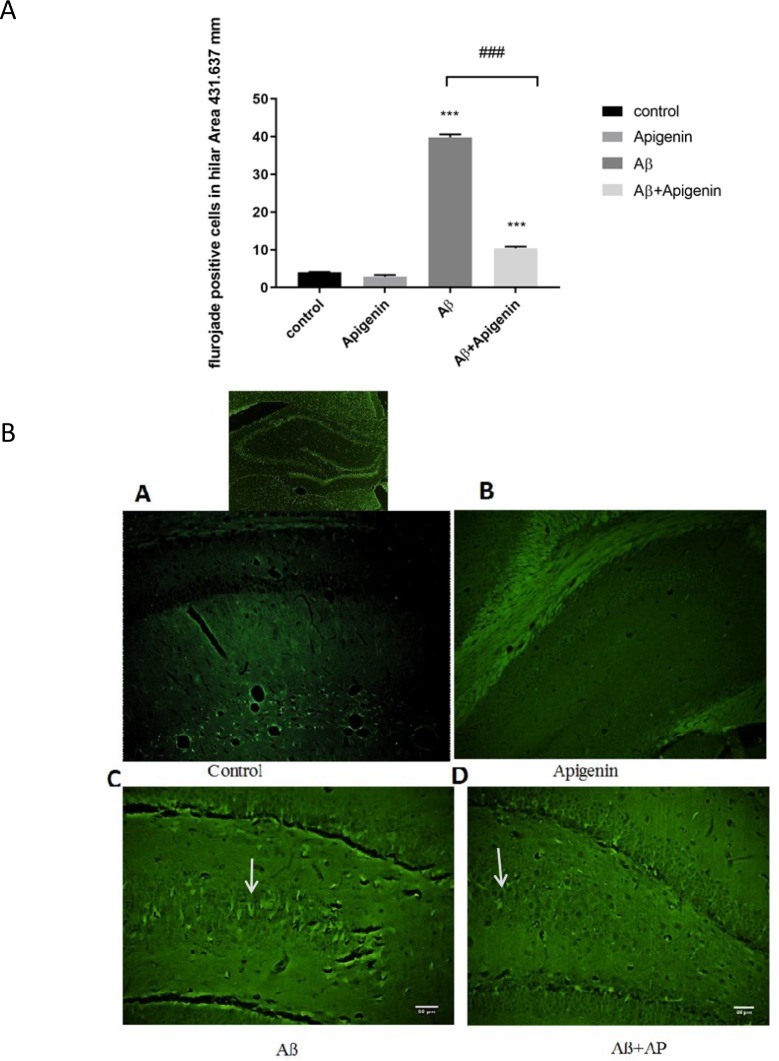
A. Representative bar graphs showing quantitative analysis of degenerative cells in the hilus area stained by Fluoro-Jade B (FJB). A significant reduction in the number of FJB-positive cells is evident following treatment with apigenin. Values are expressed as Mean±SE. ### P < 0.001 vs. amyloid beta (Aβ) group (n=4). B. a. Representative FJB-labeled sections taken from the control b), apigenin; c. Aβ d. and Aβ+apigenin groups 30 days after surgery. No FJB-positive cells were apparent in the control tissue. FJB-positive cells were evident in the Aβ group (indicated by arrows). FJB labeling was significantly decreased after treatment with apigenin (d). Scale bar=50 μm (a–d) (n=4).

Neuronal loss and neurodegeneration were assessed by Nissl and Fluoro-Jade B staining. Apigenin treatment significantly reduced a significant necrotic cell death caused by Aβ in hilus [(F _3,15_ =118.86; P<0.001) (Figure 2 A & B)]. Neurodegeneration was studied in the hilus area using Fluoro-Jade B treatment, and apigenin markedly reduced the number of degenerative neurons [(F _3,15_ =1.110; P<0.001) (Figure 3A & B)].

**Figure 4.**
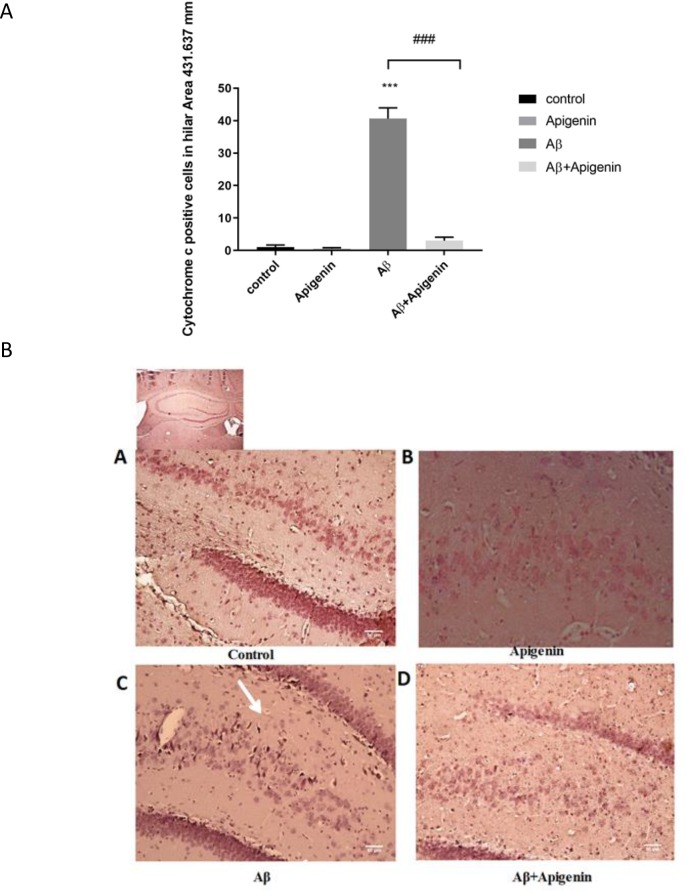
A. a. Bar chart showing quantification of immunohistochemically labeled of cytochrome c-positive cells in the hilus area of the hippocampus in the control; b. Apigenin; c. Amyloid Beta (Aβ); and d. Aβ+apigenin groups. A significant reduction in the number of cytochrome c-positive cells is evident following treatment with apigenin. Values are expressed as Mean±SE (n=4). ^###^ P<0.001 Aβ vs. Aβ+apigenin group; ^***^ Aβ vs. control group B. a. Photomicrograph of representative immunohistochemistry of cytochrome c-stained sections prepared from the control b. Apigenin; c. Amyloid beta (Aβ); and d. Aβ+Apigenin groups. Apigenin almost completely blocked the release of cytochrome c in the hilar area. Scale bar=50 μm (A–D) (n=4).

**Figure 5.**
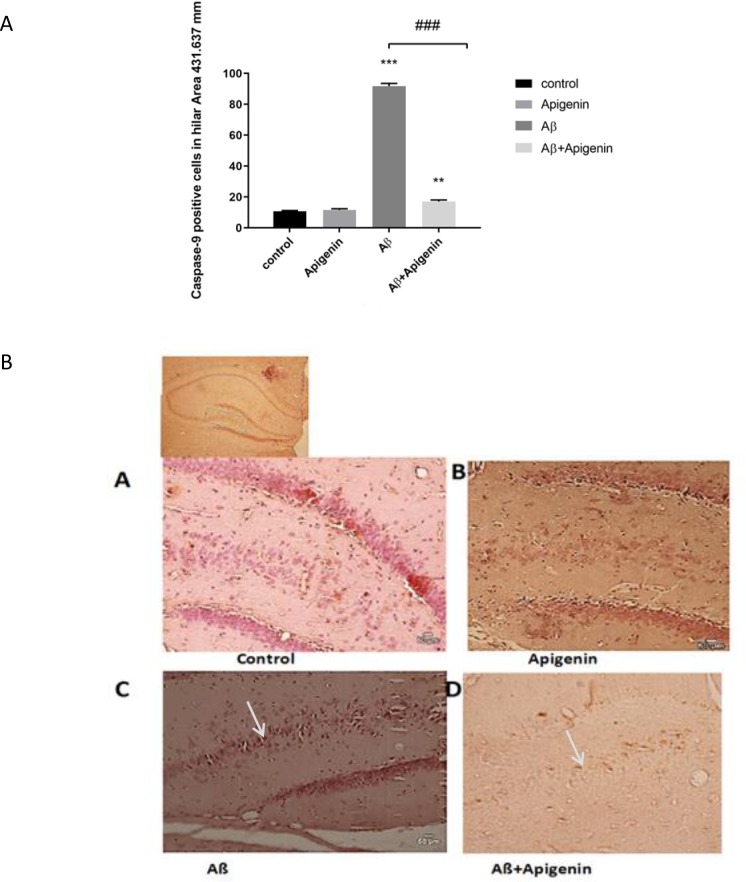
A. a. Bar chart showing quantification of immunohistochemically labeled of caspase 9-positive cells in the hilus area of the hippocampus In the control; b. Apigenin; c. Amyloid Beta (Aβ) and d. Aβ+Apigenin groups. A significant reduction in the number of caspase 9-positive cells is evident following treatment with apigenin. Values are presented as Means±SE (n=4). ^###^ P<0.001 Aβ vs. Aβ+apigenin, ^***^ Aβ vs. control group. B. a. Photomicrograph of representative immunohistochemistry of cytochrome c-stained sections prepared from the control; b. Apigenin; c. Aβ; and d. Aβ+Apigenin groups. The number of cytochrome c-positive cells significantly reduced after apigenin treatment in the hilar area. Scale bar=50μm (A–D) (n=4).

Besides, the numbers of cytochrome c and caspase 9-positive cells in the hilus area were assessed using immunohistochemistry. Apigenin intervention obviously inhibited Aβ-induced cytochrome c release [(F _3,15_ =122.419; P<0.001) (Figure 4 A & B)] and caspase 9 activation [(F _3,15_ =1.194; P<0.001) (Figure 5A & B)].

## 4. Discussion

In the present study, we evaluated the neuroprotective effects of apigenin in Aβ 25–35-induced neurotoxicity and memory impairment in a rat model of AD. Following a single ICV injection of Aβ 25–35, a massive neuronal loss was observed in the CA1, CA3, hilar, and granule cells of the dentate gyrus of the hippocampus. This massive hippocampal cell loss caused severe memory deficit. Stepanichev et al., reported a significant correlation between working memory deficit and the neuronal cell loss in the hippocampus (Stepanichev et al., 2004). Contrary to their results indicating no degeneration in CA3, our results showed neurodegeneration in the CA3 and hilus areas of the hippocampus. Many previous studies have also demonstrated the same results following the ICV injection of Aβ 25–35 (Asha & Sumathi, 2016; Mazzola, Micale, & Drago, 2003). CA1 and hilus areas are the most vulnerable areas of the hippocampus in AD. However, in this study, we just reported the results of the hilus area, because it was the most sensitive area to apigenin protective effects.

Our results revealed that apigenin treatment in AD rats could ameliorate working memory in the Y maze test. These results are in consistent with previous studies. For example, Liu et al., (2011) correlated memory amelioration effect of apigenin to an increase in acetylcholine release in the brain (Liu et al., 2011) and also Zhao et al., (2013) attributed it to the inhibition of oxidative stress and restore ERK/CREB/BDNF pathway (the most important neurotrophic pathway involved in memory). It is notable that in non-pathogenic conditions, apigenin did not affect memory. However, in the pathological conditions, like in AD, apigenin could ameliorate the working memory deficit. The probable underlying mechanisms for apigenin to ameliorate memory impairment in pathological conditions, such as AD are as follows:

Like other flavones, apigenin has antioxidative properties, by which it acts as a neuroprotective agent (Balez et al., 2016; Souza et al., 2015) and can protect hippocampal neurons against stress oxidative. It has been demonstrated that apigenin increases brain connections and stimulates the expression of synaptic markers in the brain (Souza et al., 2015). Moreover, apigenin specifically can reduce caspase 3 and caspase 7, the most important caspases in synaptic destruction (Balez et al., 2016; Souza et al., 2015). Apigenin increases the level of acetylcholine in the brain (Balez et al., 2016). Apigenin induces neurogenesis (Taupin, 2009), and even in pluripotent stem cell cultures, it increases neuronal markers, which indicate a neural differentiation stimulator for apigenin (Souza et al., 2015).

In the present study, we tried to reveal the level of neuroprotection induced by apigenin. The results demonstrated a significant reduction in neuronal loss, which can be explained by the memory amelioration in the AD group. However, apigenin, probably by using variant mechanisms, exerts its anti-amnesic effects in pathological conditions, like AD. Regarding the pivotal role of mitochondria in the pathogenesis of AD, in the final step, we tried to show the effectiveness of apigenin on inhibition of cytochrome c release from mitochondria.

In previous studies, it has been demonstrated that Aβ accumulation in the mitochondria is one of the earliest pathological conditions in AD. Therefore, any medication to cause a mitochondrial dysfunction modulation can be useful in the early stages of AD disease.

## 5. Conclusion

In the hilus area, where apigenin exerts the most protective effect, complete inhibition of cytochrome c release was observed. To the best of our knowledge, it is for the first time that the inhibitory effect of apigenin on cytochrome c release in pathological conditions, like AD, has been demonstrated. Apigenin is a protective and anti-amnesic agent in AD, which exerts its effect, at least partly through the amelioration of mitochondrial dysfunction.

## Ethical Considerations

### Compliance with ethical guidelines

All experiments were conducted according to the guide for the care and use of laboratory animals (National Institutes of Health Newsletter No. 80-23, revised 1996).
